# Hydatid Disease of the Liver. Diagnosis and Surgical Treatment

**DOI:** 10.1155/1991/45101

**Published:** 1991

**Authors:** Evaghelos Xynos, George Pechlivanides, Anastasios Tzortzinis, Antonis Papageorgiou, John S. Vassilakis

**Affiliations:** 1 The 2nd Surgical Department Athens Naval and Veterans Hospital Greece; 2 The Surgical Unit Medical School University of Crete Greece; 3 49 Ymittou Str. Cholargos Athens GR-155 61 Greece

## Abstract

A series of 155 cases of hepatic hydatid disease, occurring in 121 patients, were operated on at the Naval and Veterans Hospital of Athens. Ultrasonography and computerized axial tomography provided the preoperative diagnosis in 89 and 93 percent of the cases respectively in recent years. Thirty one percent of the cases presented with complications, the commonest of these being infection of the cyst (10 percent) and rupture of the cyst into the bile ducts (17 percent). Total cystectomy was performed in three cases and removal of the endocyst with its content in the remaining 152. The remaining cavity was either externally drained (57 cases), or filled with omentum (omentoplasty — 95 cases). External fistula and infection of the residual cavity occurred in 32 and 56 percent after simple drainage and in 4 and 2 percent respectively after omentoplasty. Differences are statistically significant (p <  0.001). Hospitalization was also significantly longer after drainage than after omentoplasty (p < 0.01). Obstructive jaundice after intrabiliary rupture of the cyst was more successfully managed after additional choledochoduodenostomy than after simple drainage of the common bile duct. Intrapericoneal recurrence of hydatid disease occurred in two cases. The conclusion of the present study is, that ultrasonography and computerized axial tomography provide an acceptable rate in the diagnosis and that omentoplasty offers a very low complication rate in the management of hydatid cystic disease of the liver.


*HPB Surgery*, 1991. Vol. 4, pp 59–67
Reprints available directly from the publisher
Photocopying permitted by license only

© 1991 Harwood Academic Publishers GmbH
Printed in the United Kingdom

### HYDATID DISEASE OF THE LIVER. DIAGNOSIS AND SURGICAL TREATMENT

EVAGHELOS XYNOS, GEORGE PECHLIVANIDES, ANASTASIOS TZORTZINIS and ANTONIS PAPAGEORGIOU
*The 2nd Surgical Department, Athens Naval and Veterans Hospital*

JOHN S. VASSILAKIS
*The Surgical Unit, Medical School, University of Crete*

*(Received 11 November 1990)*

A series of 155 cases of hepatic hydatid disease, occurring in 121 patients, were operated on at the Naval and Veterans Hospital of Athens. Ultrasonography and computerized axial tomography provided the preoperative diagnosis in 89 and 93 percent of the cases respectively in recent years. Thirty one percent of the cases presented with complications, the commonest of these being infection of the cyst (10 percent) and rupture of the cyst into the bile ducts (17 percent). Total cystectomy was performed in three cases and removal of the endocyst with its content in the remaining 152. The remaining cavity was either externally drained (57 cases), or filled with omentum (omentoplasty — 95 cases). External fistula and infection of the residual cavity occurred in 32 and 56 percent after simple drainage and in 4 and 2 percent respectively after omentoplasty. Differences are statistically significant ($p < 0.001$). Hospitalization was also significantly longer after drainage than after omentoplasty ($p < 0.01$). Obstructive jaundice after intrabiliary rupture of the cyst was more successfully managed after additional choledochoduodenostomy than after simple drainage of the common bile duct. Intrapericoneal recurrence of hydatid disease occurred in two cases. The conclusion of the present study is, that ultrasonography and computerized axial tomography provide an acceptable rate in the diagnosis and that omentoplasty offers a very low complication rate in the management of hydatid cystic disease of the liver.

Hydatid disease of the liver, caused by the tapewarm *Echinococcus Granulosus*, is endemic in countries around the Mediterranean Sea[1]. Despite the advent of chemotherapeutic agents, such as the derivatives of benzimidazole carbamate in the medical treatment of hydatidosis[2,3], surgery remains the treatment of choice[4,5]. The high complication and recurrence rates after surgery in the treatment of hepatic hydatid disease accounts for the variety of the techniques, which have been developed. Total cystectomy is indicated only in peripherally located cysts[6], while hepatic lobectomy, although ideal in eliminating the disease, is considered to be too much surgery for a benign condition[7]. The most widely applied technique is that of sterilizing the cyst, removing the endocyst (laminated and germinal layers with scolices, clear fluid and daughter cysts) and leaving intact the ectocyst[5]. The residual cavity then can be dealt with by a variety of methods, which include simple drainage, marsupialization, capitonnage, omentoplasty and capsulorrhaphy[5–9].

Address for correspondence: E. Xynos, Senior Registrar in Surgery, 49 Ymittou Str., Cholargos, GR-155 61, Athens, Greece

Herein, the experience of the authors on the diagnosis and surgical treatment of the hydatid disease of the liver over a period of 17 years is discussed.

### PATIENTS AND METHODS

From 1970 to 1986, 121 patients underwent 155 operations for the treatment of hepatic hydatid cysts at the Naval and Veterans Hospital of Athens. Seventy one of them were male and the remaining 51 were female. Their ages ranged between 19 and 78 years. In 124 cases (80 percent) the cysts were located in the right lobe, in 21 cases (13.5 percent) in the left lobe and there were 10 cases (6.5 percent) where cysts were found in both hepatic lobes. In 25 cases (16 percent) the cysts were multiple; in 11 of them (7 percent) more than two cysts were found. Preoperative diagnosis was based on symptomatology, clinical findings, laboratory data and liver scintigraphy, ultrasonography and computerized axial tomography.

### *Procedures*

Upon operation, after opening the abdominal cavity, the operative field was protected against spillage by packs, soaked in hypertonic saline solution of 15 percent. Peripheral small cysts were found in three cases. They were treated by total cystectomy. The remaining cases were treated by excision of the endocyst. In detail, the cystic fluid was removed using a closed suction system and the cystic cavity was filled with hypertonic saline solution for a few minutes. After incising the ectocyst, the endocyst containing the daughter cysts was completely removed. Debris from the ectocyst was also removed, avoiding however extensive trimming, because of the danger of haemorrhage and biliary duct erosion. Visible communications between the remaining cavity and the biliary ducts were closed with fine sutures. The remaining cavity was treated in two different ways: either by closed suction drainage, or by omentoplasty. Division of patients in those two groups was retrospective and selection of the mode the residual cavity was dealt with was based on the personal preference of each of the six surgeons involved. There was no difference concerning location and number of cysts between the two groups. At omentoplasty, the omental pedicle was prepared, avoiding the use of the whole omentum. After being brought into the cavity, the pedicle was secured to the edge of the ectocyst with sutures.

Totally calcified cysts were not treated surgically and they are not included in the study. Infected cysts were treated by simple drainage and/or omentoplasty. Cases complicated with hepatopulmonary fistula were additionally treated by wedge excision of the lung and repair of the diaphragm. Cases presented with rupture of the cyst into the biliary tree were additionally treated with exploration of the common bile duct, removal of the daughter cysts, cholecystectomy and either T-tube drainage or choledochoduodenostomy, or even endoscopic sphincterotomy in recent years.

Follow-up ranged between 3 and 19 years (mean 12.4 years) and included yearly clinical assessment, liver function tests, either liver ultrasonography or computerized axial tomography and recently endoscopic retrograde cholangiopancreatography, if necessary.

Student-t-test and chi-square test were applied in the statistical analysis of the results.

### RESULTS

### *Preoperative Symptomatology*

In the 107 uncomplicated cases the main presenting symptom was upper abdominal heaviness (82 percent), followed by upper abdominal pain (64 percent). Fourty eight cases presented with complications, the main symptoms being fever and chills (73 percent), due either to bacterial infection of the hydatid cyst (pus in the cyst at operation) or to cystic rupture into the bile ducts and cholangitis. Twenty seven cases (17 percent) presented with jaundice as a result of intrabiliary rupture of a cyst (Table 1).

**Table 1** Clinical presentation of the 155 cases.

| | *Number of cases* | *Percentage* |
|---|---|---|
| Uncomplicated Cases | 107 | 69 |
| Upper Abdominal discomfort Heaviness | 88 | 82 |
| Upper Abdominal Pain | 68 | 64 |
| Dyspepsia | 59 | 55 |
| Palpable Mass | 49 | 46 |
| Hepatomegaly | 38 | 36 |
| Complicated cases | 48 | 31 |
| Fever and Chills | 35 | 73 |
| Infection of Cyst | 16 | 33 (10) |
| Intrabiliary Rupture of Cyst | 19 | 67 (12) |
| Obstructive Jaundice | 27 | 56 (17) |
| Dyspnoea (Right Lung Compression) | 3 | 6 (2) |
| Biliptysis-Hydatidoptysis | 2 | 4(1.3) |

in parentheses: percentages on the total of 155 cases

### *Preoperative Laboratory Data*

Table 2 shows the laboratory data, from which it can be seen that the intradermal test, complement fixation test and specific antibodies against echinococcus were positive in 72, 78 and 87 percent of the cases respectively. Low titres of antibodies against echinococcus may signify a hydatid cyst containing no scolices.

The commonest abnormalities in plain abdominal X-ray film were enlargement of the liver size, areas of calcification and displacement of the diaphragm. Liver scanning with radioisotopes was the routine diagnostic test in the early years, with positive results in the 68 percent of the cases. In more recent years ultrasonography and computerized axial tomography yield positive results in 89 and 93 percent of the cases respectively. The commonest abnormalities detected by the latter tests were liver cystic formation with or without septa, calcified wall and daughter cysts. In case of intrabiliary rupture of a cyst, enlargement of the bile ducts containing daughter cysts was detected in the majority of the cases.

**Table 2** Laboratory, scintigraphic and radiological data.

| | *Number* | *Abnormal/Percentage* |
|---|---|---|
| LABORATORY DATA | | |
| Liver Function Test | | |
| Bilirubin | 155 | 32/21 |
| Alkaline Phosphatase | 152 | 41/27 |
| Transaminases | 152 | 62/41 |
| Haematological Tests | | |
| White Blood Count | 155 | 58/37 |
| Eosinophils | 155 | 69/45 |
| Prothrombin Time | 145 | 42–29 |
| Casoni Test | 137 | 99/72 |
| Weinberg Test | 131 | 102–78 |
| Antibodies against Echinococcus | 97 | 84–87 |
| RADIOLOGY | | |
| Plain X-Ray | 149 | 92/62 |
| Cholangiogram | 27 | 9/33 |
| Barium Meal | 44 | 6/14 |
| Endoscopic Retrograde Cholangio-Pancreatography | 6 | 6/100 |
| LIVER SCINTIGRAPHY | | |
| COMPUTERIZED AXIAL TOMOGRAPHY | 82 | 76/93 |
| ULTRASONOGRAPHY | 109 | 97/89 |

### *Postoperative Complications*

Tables 3 and 4 show the various procedures performed and the postoperative complications respectively. The commonest postoperative complications were infection of the residual cavity (purulent discharge from the site of drainage) and external bile fistula, occurring mostly following simple drainage of the residual cavity, with a statistically significant difference when compared with omentoplasty ($p<0.001$). More than 55 percent of the cases with external bile fistula left the hospital with drains in place. In three of them the fistula was permanent. In another four patients — all after simple drainage — external bile fistula reoccurred after discharge from hospital. In three of them obstruction of the common bile duct by daughter cysts was the cause of the recurrent fistula and these cases were treated by choledochoduodenostomy. The total complication rate in patients with simple drainage was 82 percent — significantly higher ($p < 0.001$) than that of patients who had omentoplasty (24 percent). Hospitalization period was also longer in patients with drainage (37± 14SD days) as compared to patients with omentoplasty (15± 9SD days), with a statistically significant difference ($p<0.01$).

### *Recurrences*

Intrabdominal recurrence of hydatid cysts, attributed to spillage at operation, occurred in two cases (omentum, pelvis), which were clinically manifested two and seven years respectively after the initial operation. Surgery was carried out successfully in both cases.

Recurrent cholangitis and obstructive jaundice due to residual daughter cysts in the common bile duct occurred mainly after T-tube drainage of the common bile duct rather than after choledochoduodenostomy. This was attributed to newly discharged cystic material in the biliary tree, rather than to overlooked daughter cysts in the common bile duct, which in any case, was checked by means of a peroperative cholangiogram and in some cases by peroperative choledochoscopy. The treatment of intràbiliary rupture of hydatid cyst is discussed in detail elsewhere.

**Table 3** Surgical procedures.

| | *Number* | *Percentages* |
|---|---|---|
| SIMPLE DRAINAGE | 57 | 37 |
| Uncomplicated Cases | 39 | 68 |
| Complicated Cases | 18 | 32 |
| Infected Cysts | 7 | 39 |
| with Intrabiliary Rupture | 10 | 56 |
| + T-Tube Drainage | 4 | 40 |
| + Choledochoduodenostomy | 6 | 60 |
| Miscellaneous | 1 | 5 |
| OMENTOPLASTY | 95 | 61 |
| Uncomplicated Cases | 65 | 68 |
| Complicated Cases | 30 | 32 |
| Infected cysts | 9 | 30 |
| with Intrabiliary Rupture | 17 | 56 |
| + T-Tube Drainage | 6 | 35 |
| + Choledochoduodenostomy | 11 | 65 |
| Pulmonary Lobectomy and Diaphragm Repair | 2 | 7 |
| Miscellaneous | 2 | 7 |
| TOTAL CYSTECTOMY | 3 | 2 |

**Table 4** Postoperative complications.

| *Complications* | *Drainage (n=57)* | *Omentoplasty (n=95)* |
|---|---|---|
| Infection (residual cavity) | 32(56) | 2(2) * |
| External Bile Fistula | 18(32) | 4(4) * |
| Discharged with Fistula | 10(18) | 1(1) * |
| Permanent Fistula | 3(5) | – |
| Recurrent Fistula | 2(4) | 2(2) |
| Wound Infection | 7(12) | 11(12) |
| Cardiorespiratory | 6(11) | 12(13) |
| Deep Venous Thrombosis | – | 2(2) |
| Cholangitis Δ | 3(5) | 4(4) |
| Jaundice | 3(5) | 5(5) |
| Deaths | 2(4) | 1(1) |
| Recurrent Intrabdominal Cyst | 1(2) | 1(1) |
| Morbidity | (82) | (24) * |

* statistically significant ($p < 0.001$)

Δ All cases but one occurred after T-tube drainage of the common bile duct

Percentages are in parentheses

*Intrahepatic Recurrences*

Thirty one patients out of the 121 presented with recurrent intrahepatic hydatid cysts (26 percent). Twenty eight of them had a second operation, without further evidence of intrahepatic recurrence, while the remaining three were operated upon for a third time because of a new intrahepatic recurrence.

*Mortality*

There were three postoperative deaths. One patient died of septicaemia secondary to infection of the residual cavity after simple drainage, the second one died of pancreatitis secondary to bile duct exploration (rupture of the cyst into the bile ducts) after omentoplasty and T-tube drainage of the common bile duct and the third one died of pulmonary embolism after simple drainage.

### DISCUSSION

Uncomplicated hydatid disease of the liver is usually asymptomatic or produces minimal specific symptoms[10]. Infected or ruptured in the biliary tree hydatid cysts are presented with a more pronounced clinical picture, though not specific. Suspicion of hydatid disease becomes stronger in patients from endemic areas[5]. Hydatid serology and skin tests provide false negative and false positive results in more than 15 percent of the cases respectively[5,9–14]. Liver scintigraphy identifies the intrahepatic cyst in about 85 percent of the cases[11,12], although no information can be gained about the nature of the cyst. Ultrasonography and computerized axial tomography identify the cyst in more than 90 percent of the cases[10,12,13]. Furthermore they provide characteristic images based on the nature of the cyst, including features of the wall, presence of daughter cysts, debris and septa, surrounding areas of calcification and information about the size of the bile ducts and presence of cystic material within them in the majority of the cases[5,10,12,13,15]. Ultrasonography and computerized axial tomography offered the greatest aid in preoperative diagnosis in the present series during the last eleven years.

Complicated hydatid liver disease is present in almost one third of the cases. The main complications are infection and rupture of the cyst into either the biliary tree, the peritoneal cavity or the lungs. The respective reported incidences for these complications are 8–12, 6–17, 3–11 and 2–4 percent[5,8,11–13,16,17], similar to the findings in the present series. Infection of the cyst is transmitted through communications with bile ducts, as a result of erosion by the growing cyst[11,17]. Rupture of the cyst into the biliary tree results in obstructive jaundice usually associated with cholangitis[5,8,11,12,17–19].

Dealing with the residual cavity after removal of the endocyst is a matter of controversy. As it has been previously shown by others[8,12,17], omentoplasty offers better results as compared to simple external drainage, regarding infection of the residual cavity and external bile fistula. These findings were confirmed by the results of the present comparative study. Also, simple drainage resulted in significantly longer hospitalization than omentoplasty and 18 percent of the patients with simple drainage left the hospital with a discharging fistula.

The performance of either capsulorrhaphy[9], or a closed external drainage[11] has been alternatively suggested. Both methods are liable to infection of the residual cavity in about 5 percent of the cases[9,11,20]. External bile fistula is very likely to occur after external closed drainage, if even a trivial communication of the cyst with the biliary tree has been overlooked[11,15]. Such communications occur in about 10 percent[6], or according to Weirich[21] 80 percent of cases.

Although it has been suggested that suppuration or communication of the cyst with a major bile duct precludes omentoplasty[12], the authors feel that omentoplasty can be still performed in such cases. The omentum plays an important role in the control of infection and obvious communications can be sutured[11]. Furthermore omentum seems to prevent bile leakage through small communications[17]. Omentoplasty can also be performed in cysts with a calcified wall. Total calcification of the ectocyst usually signifies a degenerated or dead hydatid cyst, which requires no surgical intervention. Such cysts must be treated only when they become complicated[11]. Simple external drainage in such cases results in a high incidence of external fistula, sine the wall of the calcified ectocyst fails to collapse[21]. We have been using the shortest omental pedicle necessary to fill the residual cavity, reserving the rest of the omentum for synchronus or possibly recurrent intrahepatic hydatid cysts.

Hydatid cysts with large communications with major bile ducts have also been drained internally by means either of cystogastrostomy or Roux-en-Y cystojejunostomy[11,21]. Such drainage is not feasible with cysts located in the dome of the liver and also access to may be made difficult in any future operation.

Most of those with T-tube drainage of the common bile duct alone for the treatment of obstructive jaundice presented with recurrent jaundice, while only one did so following choledochoduodenostomy. Furthermore three patients, who had undergone simple T-tube drainage of the common bile duct presented with recurrent external bile fistula as a result of daughter cysts remaining in the common bile duct. We believe, that for patients with liver hydatid cyst ruptured into the biliary tree, omentoplasty of the cyst, cholecystectomy and cholodochoduodenostomy prevent recurrent jaundice and external bile leak, by ensuring unobstructed bile flow to the duodenum. Recently, permanent drainage of the common bile duct can be achieved by endoscopic sphincterotomy, when indicated[22].

The incidence of recurrent hepatic hydatid cyst is not well known. Percentages of 5 to 12 have been reported, but some series include cases with intrabdominal recurrence attributed to spillage[2,9,11,13,15]. The respective percentage in the present study was as high as 24 percent. In addition, four of our patients presented with further intrahepatic recurrence. Intrahepatic recurrence of hydatid disease could be attributed either to new formation of cysts in patients living in endemic areas or to failure to detect preexisting intrahepatic small cysts during the initial operation. Also dissemination of scolices through the biliary tree and implantation elsewhere in the liver cannot be excluded as an alternative explanation[15]. We believe that the high recurrence rate in the present series is the result of new intrahepatic formation of cysts, rather than of overlooked small cysts at first place. Increased rate of recurrence is proportional to the length of follow-up[20] and may be also attributed to the use of more sophisticated diagnostic tools in recent days[15].

### *References*

1. Lewis, J.W. Jr., Koss, N. and Kerstein, M.D. (1975) A review of echinococcal disease. *Ann. Surg.* **181**, 390–396.

2. Behkti, A., Schaaps, J.P., Capron, M., Dessaint, J.P., Santoro, F. and Capron, A. (1977) Treatment of hepatic hydatid disease with mebandazole; preliminary results in 4 cases. *Br. Med. J.* **2**, 1047-1051.
3. Morris, D.L., Dykes, P.W. and Marriner, *et al.* (1985) Albentazole; objective evidence of response in human hydatid disease. *JAMA* **253**, 2053–1057.
4. Davis, A., Pawlowski, Z.S. and Dixon, H. (1986) Multicentre clinical trials of bemantazole carbamates in human echinococcosis. *Bull. World Health Org.* **64**, 383–388.
5. Langer, B. (1987) Surgical treatment of hydatid disease of the liver. *Br. J. Surg.* **74**, 237–238.
6. Pissiotis, C.A., Wander, J.V. and Condon, R.E. (1972) Surgical treatment of hydatid disease: Prevention of complications and recurrences. *Arch. Surg.* **104**, 454–459.
7. Dintsman, M., Chaimoff, C. and Wolock, Y. (1971) Surgical treatment of hydatid cyst of the liver. *Arch. Surg.* **103**, 76–78.
8. Papadimitriou, J. and Mandrekas, A. (1970) The surgical treatment of hydatid disease of the liver. *Br. J. Surg.* **57**, 431–433.
9. Ekrami, Y. (1976) Surgical treatment of hydatid disease of the liver. *Arch. Surg.* **111**, 1350–1352.
10. Harris, K.M., Morris, D.L., Tudor, R., Toghill, P. and Hardcastle, J.D. (1986) Clinical and radiological features of simple and hydatid cysts of the liver. *Br. J. Surg.* **73**, 835–838.
11. Barros, J.L. (1978) Hydatid disease of the liver. *Am. J. Surg.* **135**, 597–601.
12. Sayek, I., Yalin, R. and Sanac, Y. (1980) Surgical treatment of hydatid disease of the liver. *Arch. Surg.* **115**, 847–850.
13. Pitt, H.A., Korzelius, J. and Tompkins, R.K. (1986) Management of hepatic echinococcosis in Southern California. *Am. J. Surg.* **152**, 110–113.
14. Lass, N., Laver, Z. and Lengy, J. (1973) The immunodiagnosis of hydatid disease: Post-operative evaluation of skin test and four serological tests. *Ann. Allergy* **31**, 430–436.
15. Langer, J.C., Rose, D.B., Keystone, J.S., Taylor, B.R., and Langer, B. (1984) Diagnosis and management of hydatid disease of the liver: A 15-year North American experience. *Ann. Surg.* **199**, 412–417.
16. Mottaghian, H. and Saidi, F. (1978) Postoperative recurrence of hydatid disease. *Br. J. Surg.* **778**, 237–242.
17. Elhamel, A. and Murthy, B.S. (1986) Hepatic hydatid disease in Libya. *Br. J. Surg.* **73**, 125–127.
18. Padadimitriou, J., Tsiftsis, D. and Tountas, C. (1983) Hydatid cyst of the liver ruptured into the biliary tree. *Curr. Surg.* 339–346.
19. Alper, A., Ariogul, O., Emre, A., Uras, A. and Okten, A. (1987) Choledochoduodenostomy for intrabiliary rupture of hydatid cysts of the liver. *Br. J. Surg.* **74**, 243–245.
20. Demirci, S., Eraslan, S., Anadol, E. and Bozatli, L. (1989) Comparison of the results of different techniques in the management of hydatid cysts of the liver. *World J. Surg.* **13**, 88–91.
21. Weirich, W.L. (1979) Hydatid disease of the liver. *Am. J. Surg.* **138**, 805–808.
22. Vignote, M.L., Mino, G., de la Mata, M., de Dios, J.F. and Gomez, F. (1990) Endoscopic sphincterotomy in hepatic hydatid disease open to the biliary tree. *Br. J. Surg.* **77**, 30–1.

*(Accepted by S. Bengmark 11 November 1990)*

### INVITED COMMENTARY

Xynos and co-authors present a very large experience from one centre in surgical treatment of hydatid disease of the liver. It is a retrospective review and makes it difficult therefore to draw comparisons between treatment options described, but the information provided is useful to those who may encounter this disease in their practice. The conclusions, however, need to be critically examined in the light of other information available in the literature.

The authors' approach is generally conservative, avoiding complete cystic excision, or major hepatic resection, and they have a respectably low operative mortality. I agree with this general approach, however, am surprised at the high recurrence rate (27%) of intrahepatic and peritoneal disease, as well as persistent jaundice and cholangitis due to "newly discharged cystic material into the biliary tree". These findings suggest that the technique described has not succeeded in completely removing all of the livering parasite. This technique therefore, should be re-examined. Our own practice includes using protective plastic sheeting to avoid spill into the peritoneal cavity[1] and using this technique we have seen no peritoneal recurrences. Our management of the cavity itself does not rely on scolicidal irrigations, but rather on meticulous mechanical removal of the living parasite including all fluid, cyst lining, daughter cysts, and scolices by suction, sponges, and curettage. Only when all this material has been removed is scolicidal fluid used in the cavity to treat any residual invisible scolices. Our recurrence rate and that reported by others in the literature is less than 10%[2].

The authors correctly observe that external drainage seems to be associated with a higher incidence of infection. Whether omentoplasty is responsible for lowering the infection rate in those not drained is not proven by the data they have presented. We also have observed a lower infection rate when drains are omitted, without the necessity of using omentoplasty[3].

Finally, the authors correctly identify the common occurrence of biliary communications with or without cholangitis and jaundice. It has been well shown that biliary obstruction from cyst material can be managed by ERCP and papillotomy prior to operation for the cyst disease itself[4], and this approach should be emphasized rather than either common duct exploration or choledochoduodenoscopy.

*References*

1. Cohen, Z., Stone, R.M., and Langer, B. (1976) Surgical treatment of hydatid disease of the liver. *Can. J. of Surg.* **19**, 416–420.
2. Langer, B. (1987) Surgical treatment of hydatid disease of the Liver. *Br. J. Surg.* **74**, 237–238.
3. Langer, J.C., Rose, D.B., Keystone, J.S., Taylor, B.R., and Lanber, B. (1984) Diagnosis and management of hydatid disease of the liver. A 15-year North American Experience. *Ann. Surg.* **199**, 412–417.
4. Ponchon, T., Bory, R., Chavaillon, A. (1987). Endoscopic retrograde cholangiography and sphincterotomy for complicated hepatic hydatid cysts. *Endoscopy* **19**, 174–177.

Bernard Langer
Department of Surgery
University of Toronto
The Banting Institute
Toronto M5G 1L5
Canada

